# High-quality-draft genome sequence of the yellow-pigmented flavobacterium *Joostella marina* type strain (En5^T^)

**DOI:** 10.4056/sigs.3537045

**Published:** 2013-04-15

**Authors:** Erko Stackebrandt, Olga Chertkov, Alla Lapidus, Matt Nolan, Susan Lucas, Cliff Han, Jan-Fang Cheng, Roxanne Tapia, Lynne A. Goodwin, David Bruce, Sam Pitluck, Konstantinos Liolios, Konstantinos Mavromatis, Ioanna Pagani, Natalia Ivanova, Natalia Mikhailova, Marcel Huntemann, Amrita Pati, Amy Chen, Krishna Palaniappan, Manfred Rohde, Brian J. Tindall, Markus Göker, Tanja Woyke, John C. Detter, James Bristow, Jonathan A. Eisen, Victor Markowitz, Philip Hugenholtz, Hans-Peter Klenk, Nikos C. Kyrpides

**Affiliations:** 1 Leibniz-Institute DSMZ - German Collection of Microorganisms and Cell Cultures, Braunschweig, Germany; 2 DOE Joint Genome Institute, Walnut Creek, California, USA; 3 Los Alamos National Laboratory, Bioscience Division, Los Alamos, New Mexico, USA; 4 Biological Data Management and Technology Center, Lawrence Berkeley National Laboratory, Berkeley, California, USA; 5 HZI – Helmholtz Centre for Infection Research, Braunschweig, Germany; 6 University of California Davis Genome Center, Davis, California, USA; 7 Australian Centre for Ecogenomics, School of Chemistry and Molecular Biosciences, The University of Queensland, Brisbane, Australia

**Keywords:** Gram-negative, non-motile, aerobic, mesophile, *Flavobacteriaceae*, *Bacteroidetes*, GEBA

## Abstract

At present, *Joostella marina* Quan *et al*. 2008 is the sole species with a validly published name in the genus *Joostella*, family *Flavobacteriacae*, phylum *Bacteriodetes*. It is a yellow-pigmented, aerobic, marine organism about which little has been reported other than the chemotaxonomic features required for initial taxonomic description. The genome of *J. marina* strain En5^T^ complements a list of 16 *Flavobacteriaceae* strains for which complete genomes and draft genomes are currently available. Here we describe the features of this bacterium, together with the complete genome sequence, and annotation. This is the first member of the genus *Joostella* for which a complete genome sequence becomes available. The 4,508,243 bp long single replicon genome with its 3,944 protein-coding and 60 RNA genes is part of the *** G****enomic* ***E****ncyclopedia of* ***Bacteria**** and* ***Archaea***** project.

## Introduction

Strain En5^T^ (= DSM 19592 = KCTC 12518 = CGMCC 1.6973) is the type strain of *Joostella marina* [1], which is the type species of the monospecific genus *Joostella* that was named after P.J. Jooste, who first proposed the family *Flavobacteriaceae* [1]. A second species name, ‘*Joostella atrarenae*’ [2] has been effectively published but not yet appeared on a validation list. *J. marina* was isolated by dilution-plating on marine agar 2216 (Difco) from coastal seawater in the East Sea of Korea. The phylogenetically neighboring genera are *Zhouia* [3] and *Galbibacter* [4]. Here we present a summary classification and a set of features for *J. marina* En5^T^ together with the description of the complete genomic sequencing and annotation. The genome of strain En5^T^ complements a list of 16 *Flavobacteriaceae* [5,6] strains for which complete genomes and draft genomes are already available.

## Classification and features

### 16S rRNA gene sequence analysis

A representative genomic 16S rRNA gene sequence of *J. marina* En5^T^ was compared using NCBI BLAST [7,8] under default settings (e.g., considering only the high-scoring segment pairs (HSPs) from the best 250 hits) with the most recent release of the Greengenes database [9] and the relative frequencies of taxa and keywords (reduced to their stem [10]) were determined, weighted by BLAST scores. The most frequently occurring genera were *Cellulophaga* (15.8%), *Aquimarina* (14.2%), *Flavobacterium* (10.7%), *Formosa* (6.9%) and *Psychroserpens* (6.1%) (123 hits in total). Regarding the single hit to sequences from members of *J. marina*, the average identity within HSPs was 100.0%, whereas the average coverage by HSPs was 99.0%. Among all other species, the one yielding the highest score was *'Venteria marina'* (DQ097522), which corresponded to an identity of 100.0% and an HSP coverage of 99.0%. (Note that the Greengenes database uses the INSDC (= EMBL/NCBI/DDBJ) annotation, which is not an authoritative source for nomenclature or classification.). The record for DQ097522 was, however, subsequently removed from Genbank at the submitter's request, because the source organism could not be confirmed. The highest-scoring environmental sequence was DQ490025 (Greengenes short name 'Microbial life ridge flank crustal fluids clone ODP-33B-02'), which showed an identity of 99.7% and an HSP coverage of 100.0%. The most frequently occurring keywords within the labels of all environmental samples which yielded hits were 'marin' (5.2%), 'water' (3.7%), 'microbi' (3.1%), 'sea' (2.9%) and 'north' (2.0%) (127 hits in total). Environmental samples which yielded hits of a higher score than the highest scoring species were not found.

**Figure 1:**
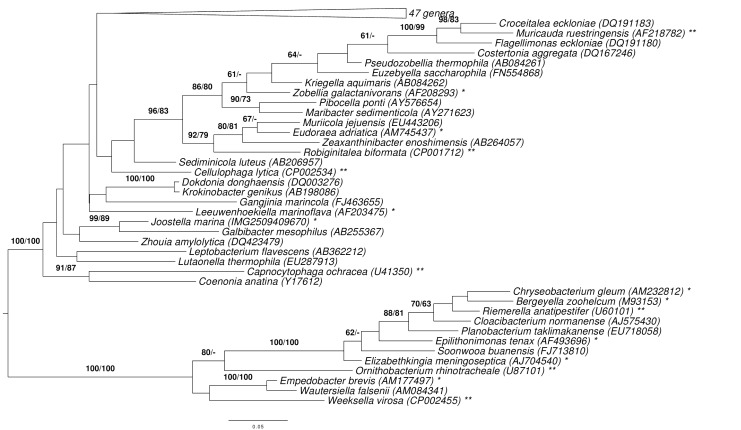
Phylogenetic tree highlighting the position of *J. marina* relative to the type strains of the type species of the other genera within the family *Flavobacteriaceae*. The tree was inferred from 1,370 aligned characters [11,12] of the 16S rRNA gene sequence under the maximum likelihood (ML) criterion [13]. Rooting was done initially using the midpoint method [14] and then checked for its agreement with the current classification (Table 1). The branches are scaled in terms of the expected number of substitutions per site. Numbers adjacent to the branches are support values from 600 ML bootstrap replicates [15] (left) and from 1,000 maximum-parsimony bootstrap replicates [16] (right) if larger than 60%. Lineages with type strain genome sequencing projects registered in GOLD [17] are labeled with one asterisk, those also listed as 'Complete and Published' with two asterisks (see CP003283 for *Ornithobacterium rhinotracheale* and [18-23]).

Figure 1 shows the phylogenetic neighborhood of *J. marina* in a 16S rRNA based tree. The sequences of the three identical 16S rRNA gene copies in the genome do not differ from the previously published 16S rDNA sequence (EF660761).

**Table 1:** Classification and general features of *J. marina* En5^T^ according to the MIGS recommendations [24].

**MIGS ID**	**Property**	**Term**	**Evidence code**
	Current classification	Domain *Bacteria*	TAS [25]
		Phylum *Bacteroidetes*	TAS [26,27]
		Class *Flavobacteriia*	TAS [28-30]
		Order *Flavobacteriales*	TAS [27,31]
		Family *Flavobacteriaceae*	TAS [5,6,32,33]
		Genus *Joostella*	TAS [1]
MIGS-7		Species *Joostella marina*	TAS [1]
MIGS-12	Subspecific genetic lineage (strain)	En5^T^	TAS [1]
	Reference for biomaterial	Quan *et al*. 2008	TAS [1]
	Gram stain	negative	TAS [1]
	Cell shape	rod-shaped	TAS [1]
	Motility	non-motile	TAS [1]
	Sporulation	non-sporulating	TAS [1]
	Temperature range	10-37°C	TAS [1]
	Optimum temperature	30°C	TAS [1]
MIGS-22	Salinity	0-15% NaCl, optimally 1-3% NaCl	TAS [1]
	Relationship to oxygen	obligate aerobe	TAS [1]
	Carbon source	monosaccarides	TAS [1]
MIGS-6	Energy metabolism	not reported	
MIGS-6.2	Habitat	mud	TAS [1]
MIGS-15	pH	optimum 5.3 - 7.6	TAS [1]
MIGS-14	Biotic relationship	free living	TAS [1]
MIGS-16	Known pathogenicity	not reported	
MIGS-18	Specific host	none	NAS
	Health status of host	not reported	
MIGS-19	Biosafety level	1	TAS [34]
MIGS-23.1	Trophic level	not reported	
MIGS-4	Isolation	coastal seawater	TAS [1]
MIGS-5	Geographic location	East Sea of Korea	TAS [1]
MIGS-4.1	Time of sample collection	May 2007	NAS
MIGS-4.2	Latitude	not reported	
MIGS-4.3	Longitude	not reported	
MIGS-4.4	Depth	100 m	TAS [1]
	Altitude	- 100 m	TAS [1]

Evidence codes - TAS: Traceable Author Statement (i.e., a direct report exists in the literature); NAS: Non-traceable Author Statement (i.e., not directly observed for the living, isolated sample, but based on a generally accepted property for the species, or anecdotal evidence). Evidence codes are from the Gene Ontology project [35].

### Morphology and physiology

**Figure 2:**
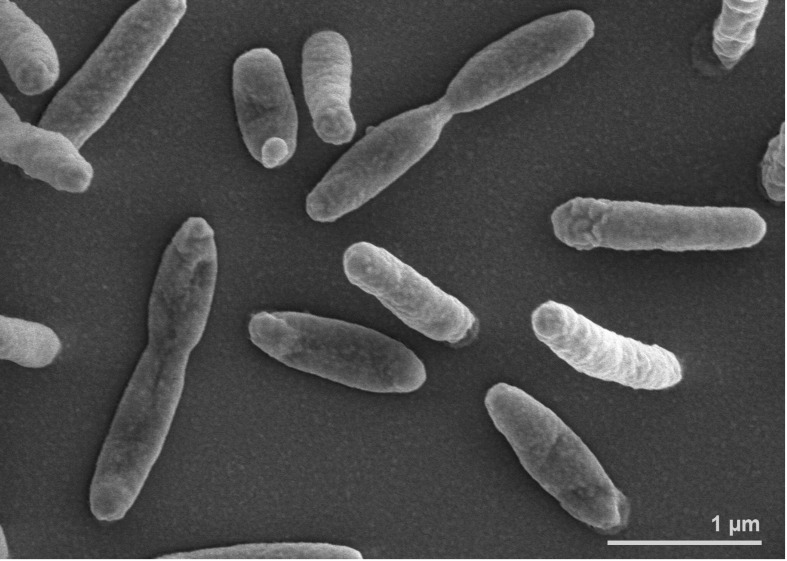
Scanning electron micrograph of *J. marina* En5^T^

The rod-shaped cells of *strain* En5^T^ (0.2-0.3 µm wide and 1.0-2.0 µm long) stain Gram-negative [1] (Figure 2). Flexirubin-type pigments are not formed and gliding motility is absent. The optimal NaCl concentration for growth is 1-3% but cells can grow in up to 15% NaCl. Optimal growth temperature is 30°C and no growth is observed at 4°C or at 42°C. Growth occurs at pH 5.3-10.5 with an optimum between pH 5.3 and 7.6. The organism is oxidase- and catalase-positive and strictly aerobic. Nitrate and nitrite are not reduced. Starch, aesculin and Tween 80 are hydrolyzed, but agar, casein and gelatin are not hydrolyzed. Glucose, sucrose, arabinose, mannose and maltose are utilized as sole carbon source while mannitol, N-acetylglucosamine, gluconate, caprate, adipate, malate, citrate and phenylacetate are not utilized. Acid is produced from cellobiose, but not from glucose. Cells are positive for α-glucosidase, β-glucosidase, β-galactosidase, α-mannosidase, alkaline phosphatase, acid phosphatase, esterase (C4), esterase lipase (C8), leucine arylamidase, valine arylamidase, cystine arylamidase, trypsin, naphthol-AS-BI-phosphohydrolase and N-acetyl-β-glucosaminidase and negative for the other enzyme activities tested by the API ZYM (bioMérieux) panel [1].

### Chemotaxonomy

Major fatty acids (>10% of total) are branched-chain acids *iso*-C_15:0_, *iso*-C_17:0 3-OH_ and *iso*-C_17:1_ *_ω_*_9_*_c_* and an unidentified fatty acid (ECL 13.566); minor amounts (>5%-<10%) are *iso*-C_15:1_ and summed feature 3 comprising C_16:1_ *_ω_*_7_*_c_* and/or *iso*-C_15:0 2-OH_. It should be noted that the original paper indicates that the fatty acid composition was determined using the MIDI system and in the peak naming tables *iso*-C_15:1_ is usually not listed without the addition of further information (e.g. *iso*-C_15:1_ F, *iso*-C_15:1_ G, *iso*-C_15:1_ H, with the capital letters indicating different isomers where the location of the double bond is not determined). Herzog *et al.* [36], have indicated that the fatty acid listed as *iso*-C_17:1_ *_ω_*_9_*_c_* may be incorrectly annotated in the MIDI system. Furthermore the resolution of summed feature 3 into C_16:1_ *_ω_*_7_*_c_* and/or *iso*-C_15:0 2-OH_ is also significant in understanding the membrane structure/function as well as the evolution of the underlying biochemical pathways, since the synthesis of 2-OH fatty acids requires a specific enzyme, whereas the synthesis of unsaturated fatty acids (with different positions of unsaturation) also requires a specific set of enzymes. MK-6 is the major respiratory quinone. The DNA G+C content was initially reported with 30.1 mol% [1], much lower than the 33.6% inferred from the genome sequence (see in third table). No information is available for the peptidoglycan composition as this feature is not listed as a minimal standard for the descriptions of novel *Flavobacteriaceae* species [33]. No data is available on the polar lipid composition.

## Genome sequencing and annotation

### Genome project history

This organism was selected for sequencing on the basis of its phylogenetic position [37], and is part of the *** G****enomic* ***E****ncyclopedia of* ***Bacteria**** and* ***Archaea***** project [38]. The genome project is deposited in the Genomes OnLine Database [17] and the complete genome sequence is deposited in GenBank. Sequencing, finishing and annotation were performed by the DOE Joint Genome Institute (JGI) using state of the art sequencing technology [39]. A summary of the project information is shown in Table 2.

**Table 2:** Genome sequencing project information

MIGS ID	Property	Term
MIGS-31	Finishing quality	Improved high quality draft
MIGS-28	Libraries used	Two genomic libraries: one 454 PE library (8 kb insert size), one Illumina library
MIGS-29	Sequencing platforms	Illumina GAii, 454 GS FLX Titanium
MIGS-31.2	Sequencing coverage	1,149.8 × Illumina; 8.6 × pyrosequence
MIGS-30	Assemblers	Newbler version 2.3-PreRelease-6/30/2009, Velvet 1.0.13, phrap version 1.080812
MIGS-32	Gene calling method	Prodigal 1.4, GenePRIMP
	INSDC ID	AJUG00000000
	GenBank Date of Release	May 4, 2012
	GOLD ID	Gi05349
	NCBI project ID	65069
	Database: IMG	2509276026
MIGS-13	Source material identifier	DSM 19592
	Project relevance	Tree of Life, GEBA

### Growth conditions and DNA isolation

*J. marina* strain En5^T^, DSM 19592, was grown in DSMZ medium 514 (Bacto Marine Broth, DIFCO 2216) [40] at 28°C. DNA was isolated from 1-1.5 g of cell paste using Jetflex Genomic DNA Purification Kit (GENOMED 600100) following the standard protocol as recommended by the manufacturer with modification but with additional 10 µl proteinase K digestion for cell lysis (40 min incubation at 58°C). DNA is available through the DNA Bank Network [41].

### Genome sequencing and assembly

The genome was sequenced using a combination of Illumina and 454 sequencing platforms. All general aspects of library construction and sequencing can be found at the JGI website [42]. Pyrosequencing reads were assembled using the Newbler assembler (Roche). The initial Newbler assembly, consisting of 240 contigs in 6 scaffolds, was converted into a phrap [43] assembly by making fake reads from the consensus, to collect the read pairs in the 454 paired end library. Illumina GAii sequencing data (5,373.5 Mb) was assembled with Velvet [44] and the consensus sequences were shredded into 1.5 kb overlapped fake reads and assembled together with the 454 data. The 454 draft assembly was based on 76.9 Mb 454 draft data and all of the 454 paired end data. Newbler parameters are -consed -a 50 -l 350 -g -m -ml 21. The Phred/Phrap/Consed software package [43] was used for sequence assembly and quality assessment in the subsequent finishing process. After the shotgun stage, reads were assembled with parallel phrap (High Performance Software, LLC). Possible mis-assemblies were corrected with gapResolution [42], Dupfinisher [45], or by sequencing cloned bridging PCR fragments with subcloning. Gaps between contigs were closed by editing in Consed, by PCR and by Bubble PCR primer walks (J.-F. Chang, unpublished). A total of 193 additional reactions and one shatter library were necessary to close some gaps and to raise the quality of the final contigs. Illumina reads were also used to correct potential base errors and increase consensus quality using a software Polisher developed at JGI [46]. The error rate of the final genome sequence is less than 1 in 100,000. Together, the combination of the Illumina and 454 sequencing platforms provided 1,158.4 × coverage of the genome. The final assembly contained 219,876 pyrosequence and 68,081,556 Illumina reads.

### Genome annotation

Genes were identified using Prodigal [47] as part of the DOE-JGI genome annotation pipeline [48], followed by a round of manual curation using the GenePRIMP pipeline [49]. The predicted CDSs were translated and used to search the National Center for Biotechnology Information (NCBI) non-redundant database, UniProt, TIGR-Fam, Pfam, PRIAM, KEGG, COG, and InterPro databases. Additional gene prediction analysis and functional annotation was performed within the Integrated Microbial Genomes - Expert Review (IMG-ER) platform [50].

## Genome properties

**Figure 3:**
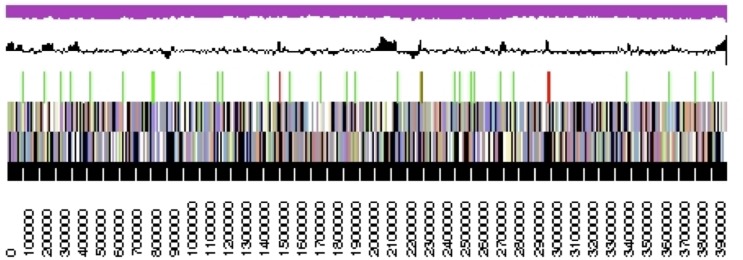
Graphical maps of the largest, 3.96 Mbp long, scaffold. From bottom to top: Genes on forward strand (color by COG categories), Genes on reverse strand (color by COG categories), RNA genes (tRNAs green, rRNAs red, other RNAs black), GC content, GC skew (purple/olive).

The genome statistics are provided in Table 3 and Figure 3. The improved-high-quality-draft genome consists of two scaffolds with a length of 3,959,031 bp and 558,212 bp, respectively, and a G+C content of 33.6%. Of the 4,004 genes predicted, 3,944 were protein-coding genes, and 60 RNAs; 86 pseudogenes were also identified. The majority of the protein-coding genes (69.4%) were assigned a putative function while the remaining ones were annotated as hypothetical proteins. The distribution of genes into COGs functional categories is presented in Table 4.

**Table 3:** Genome Statistics

**Attribute**	**Value**	**% of Total**
Genome size (bp)	4,508,243	100.00
DNA coding region (bp)	3,886,653	86.21
DNA G+C content (bp)	1,514,507	33.59
Number of scaffolds	2	
Extrachromosomal elements	unknown	
Total genes	4,004	100.00
RNA genes	60	1.50
rDNA operons	3	
tRNA genes	45	1.12
Protein-coding genes	3,944	98.50
Pseudo genes	86	2.15
Genes with function prediction	2,777	69.36
Genes in paralog clusters	2,029	50.67
Genes assigned to COGs	2,678	66.88
Genes assigned Pfam domains	3,099	77.40
Genes with signal peptides	1,055	26.35
Genes with transmembrane helices	940	23.48
CRISPR repeats	1	

**Table 4:** Number of genes associated with the general COG functional categories

**Code**	**Value**	**% age**	**Description**
J	153	5.29	Translation, ribosomal structure and biogenesis
A	0	0.00	RNA processing and modification
K	203	7.01	Transcription
L	225	7.77	Replication, recombination and repair
B	0	0.00	Chromatin structure and dynamics
D	23	0.79	Cell cycle control, cell division, chromosome partitioning
Y	0	0.00	Nuclear structure
V	50	1.73	Defense mechanisms
T	123	4.25	Signal transduction mechanisms
M	222	7.67	Cell wall/membrane/envelope biogenesis
N	6	0.21	Cell motility
Z	0	0.00	Cytoskeleton
W	0	0.00	Extracellular structures
U	62	2.14	Intracellular trafficking, secretion, and vesicular transport
O	119	4.11	Posttranslational modification, protein turnover, chaperones
C	133	4.60	Energy production and conversion
G	189	6.53	Carbohydrate transport and metabolism
E	211	7.29	Amino acid transport and metabolism
F	64	2.21	Nucleotide transport and metabolism
H	144	4.98	Coenzyme transport and metabolism
I	97	3.35	Lipid transport and metabolism
P	206	7.12	Inorganic ion transport and metabolism
Q	50	1.73	Secondary metabolites biosynthesis, transport and catabolism
R	346	11.96	General function prediction only
S	268	9.26	Function unknown
-	1326	33.12	Not in COGs
